# Spatial genetic structure within populations and management implications of the South American species *Acacia aroma* (Fabaceae)

**DOI:** 10.1371/journal.pone.0192107

**Published:** 2018-02-01

**Authors:** Carolina Pometti, Cecilia Bessega, Ana Cialdella, Mauricio Ewens, Beatriz Saidman, Juan Vilardi

**Affiliations:** 1 Universidad de Buenos Aires, Facultad de Ciencias Exactas y Naturales, Departamento Ecología, Genética y Evolución, Genética de Especies Leñosas (GEEL), Buenos Aires, Argentina; 2 CONICET-Universidad de Buenos Aires, Instituto de Ecología, Genética y Evolución (IEGEBA), Buenos Aires, Argentina; 3 IBODA, CONICET, San Isidro, Buenos Aires, Argentina; 4 Estación Experimental Fernández-UCSE (Convenio Provincia Sgo del Estero- Universidad Católica Sgo del Est.), Departamento de Robles, Santiago del Estero, Argentina; Consiglio Nazionale delle Ricerche, ITALY

## Abstract

The identification of factors that structure intraspecific diversity is of particular interest for biological conservation and restoration ecology. All rangelands in Argentina are currently experiencing some form of deterioration or desertification. *Acacia aroma* is a multipurpose species widely distributed throughout this country. In this study, we used the AFLP technique to study genetic diversity, population genetic structure, and fine-scale spatial genetic structure in 170 individuals belonging to 6 natural Argentinean populations. With 401 loci, the mean heterozygosity (*H*_*E*_ = 0.2) and the mean percentage of polymorphic loci (*PPL* = 62.1%) coefficients indicated that the genetic variation is relatively high in *A*. *aroma*. The analysis with STRUCTURE showed that the number of clusters (*K*) was 3. With Geneland analysis, the number of clusters was *K* = 4, sharing the same grouping as STRUCTURE but dividing one population into two groups. When studying SGS, significant structure was detected in 3 of 6 populations. The neighbourhood size in these populations ranged from 15.2 to 64.3 individuals. The estimated gene dispersal distance depended on the effective population density and disturbance level and ranged from 45 to 864 m. The combined results suggest that a sampling strategy, which aims to maintain a considerable part of the variability contained in natural populations sampled here, would include at least 3 units defined by the clusters analyses that exhibit particular genetic properties. Moreover, the current SGS analysis suggests that within the wider management units/provinces, seed collection from *A*. *aroma* should target trees separated by a minimum distance of 50 m but preferably 150 m to reduce genetic relatedness among seeds from different trees.

## Introduction

The spatial structure of the genomic variation among natural populations constitutes a central topic in evolutionary biology. The structure is primarily influenced by the population density, breeding system, and environmental heterogeneity, among other factors. For plants, the ability to extend the geographical distribution and maintain genetic variability within populations depends on the gene flow mediated by seed movement and pollen dispersal [1, 2]. These mechanisms influence the structuration of genetic diversity within and between populations, which is usually referred to as spatial genetic structure (SGS) [3]. The spatial distribution of individuals within a population is a considerable determinant of population genetic structure, and this is affected by dispersal processes. Consequently, studying the causes of SGS in plant species, in particular in *Acacia aroma* due to its ecological and economic importance in South America, is useful in conservation and management strategies for maintaining genetic diversity, particularly in stages of rapid habitat degradation [4].

Habitat fragmentation and degradation may reduce the size and increase the spatial isolation of plant populations. This could lead to increased random genetic drift, elevated inbreeding and reduced gene flow [4, 5, 6]. Therefore, when habitat degradation is occurring, it is important to measure baseline SGS to inform management decisions aimed at maintaining genetic variation [7].

*Acacia s*.*l*. is the second largest genus in the family Leguminosae (Fabaceae) and currently comprises more than 1,450 species in three subgenera. Additionally, in many dryland areas, *Acacia s*.*l*. is the dominant shrub or tree on which humans and animals depend, for example *A*. *nilotica* in Africa, *A*. *farnesiana* in Mexico, and *A*. *aroma* in Argentina, Bolivia and Peru [8]. *Acacia aroma* Gillies ex Hook. & Arn. is an ecologically and economically valuable species facing considerable habitat degradation and is well distributed throughout the Chaco Region, where it is commonly known as “aromito” or “tusca”. This species is an outcrosser; it is pollinated by bees [9], and its seeds are dispersed by native mammals, livestock, goats and horses [10]. The deterioration of landscapes in Argentina began two centuries ago, and although the human population was very small, overgrazing without regard for environmental impact was standard practice. The ecosystems were fragile and prone to extensive damage, and consequently, Argentine rangelands underwent desertification. The Chaco Region in Northwest Argentina experienced severe forest exploitation and overgrazing. Cattle production progressively expanded in this area, and due to overgrazing, resulted in a shift from grasses to shrubs and saplings according to the availability. Later, goats replaced cattle as they were able to eat almost any plant, thereby removing the community from the climax stage. Currently in this region, some *Acacia s*.*l*. species occur together with other legumes, and they provide pods for forage, lumber and medicinal products [11].

*A*. *aroma* grows as a tree or shrub 4–6 m in height, which is economically and ecologically important because of multiple uses extending from its roots to its pollen. From an ecological point of view, the roots are good nitrogen fixers, and the fruits and leaves provide forage for cattle and goats. Economically, the fruits and bark are rich in tannins, the flowers are useful in the perfume industry, the seeds have medicinal uses and the pollen is used in honey production [11]. Currently, there are few studies on population structure and genetic diversity in natural populations of *A*. *aroma*.

Thus, our main objectives are to analyse the genetic diversity and structure and characterize fine-scale spatial genetic structure (SGS) in five natural populations and a remnant of *A*. *aroma* in the Chaco Biogeographical Region in Northwest Argentina, each with different levels of disturbance. The results of this study can be used to facilitate the sustainable management of *A*. *aroma*, and this methodology can be extended to other *Acacia* species.

## Materials and methods

### Plant material

**Fig 1:**
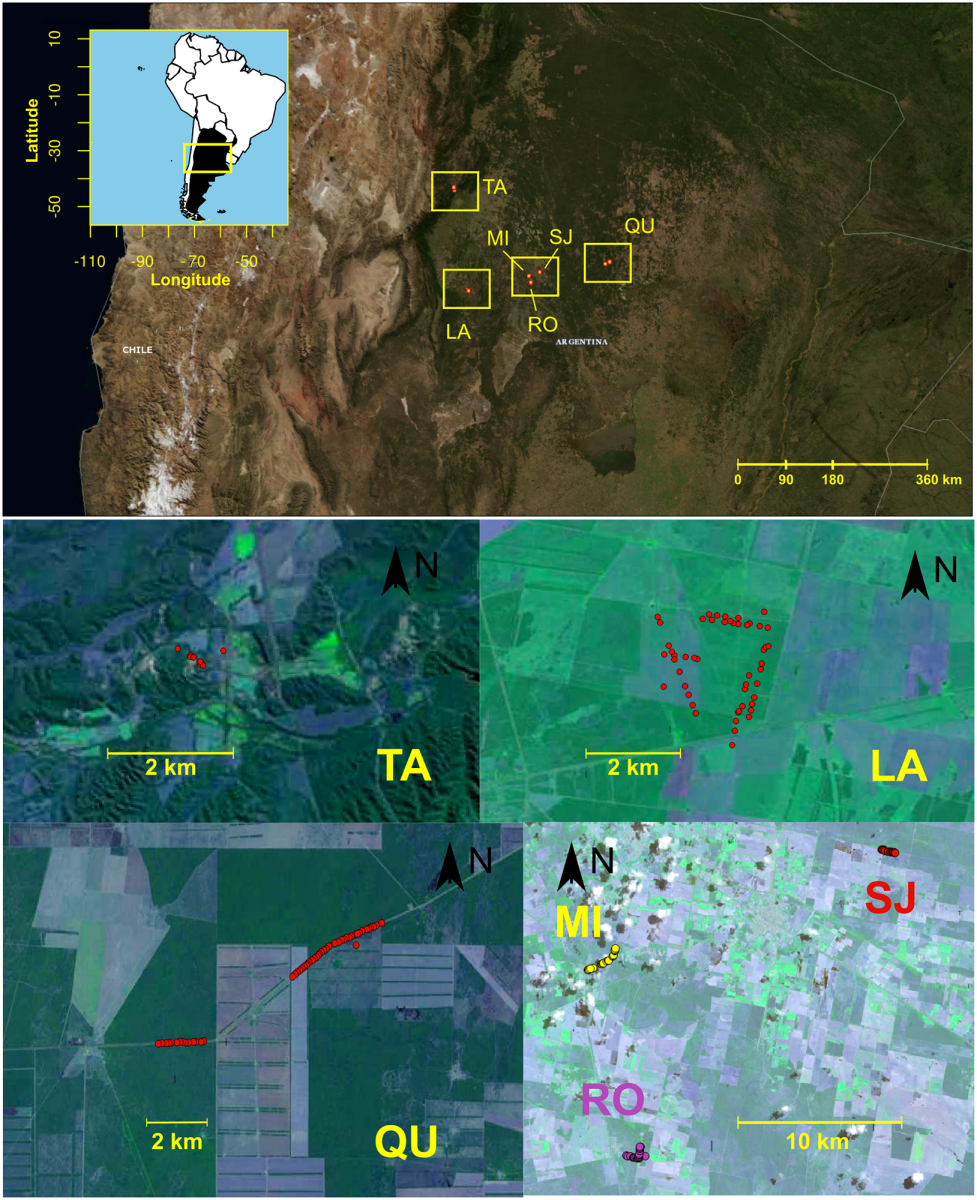
Map of Argentina showing the region and amplified zones where populations of *A*. *aroma* were sampled. Population abbreviations: LA: Lavalle, MI: Mili, QU: Quimilí, RO: Robles, SJ: San José, TA: Tapia. Base maps were obtained from http://viewer.nationalmap.gov/viewer/ and http://eros.usgs.gov/.

Young, fully grown, healthy leaves of *Acacia aroma* plants were collected in the Chaco Biogeographical Region in Northwest Argentina (Table 1 and Fig 1) and maintained in bags with silica gel prior to DNA extraction. A total of six sites were sampled, each with different levels of disturbance. No specific permissions were required in the field locations where samples were taken, as they did not involve endangered or protected species and were not on private lands. The number of sampled individuals ranged from 20 to 50 adults per site. Of these individuals, 170 (95%) yielded clear amplifications allowing for population analysis (Table 1). The sampling strategy used here covered between 70 and 100% of the adult individuals at each site, due to the small size of the populations. The population of Lavalle (LA) is the most degraded from anthropogenic activities, and currently, almost all of the area is deforested; Quimilí (QU) is located in a highly disturbed area, and its distribution is patchy; Tapia (TA) is a remnant of only 20 individuals, surrounded by soy plantations; Robles (RO) extends on both sides of a road impacted by a high level of traffic on this route; and San José (SJ) and Mili (MI) are the most pristine populations among the sampled sites.

**Table 1:** Populations of *Acacia aroma* sampled in this study and the summary of genetic diversity based on 401 neutral AFLP loci. N = sample size amplified with the AFLP technique; *PPL* = percentage of polymorphic loci; *H*_*E*_ = genetic diversity equivalent to expected heterozygosity under panmixia; S.E. (*H*_*E*_) = standard error of *H*_*E*_.

Population	Population code	Longitude (W)	Latitude (S)	N	*PLP* (%)	*H*_*E*_	S.E.(*H*_*E*_)
Lavalle	LA	65° 2′43.30"	28° 9′39.70"	50	60.3	0.21	0.01
Mili	MI	63°59′31.98"	27°56′31.50"	20	60.6	0.21	0.01
Quimilí	QU	62°38′26.04"	27°44′22.38"	50	67.1	0.23	0.01
Robles	RO	63°58′59.76"	28° 3′12.78"	22	65.8	0.21	0.01
San José	SJ	63°49′54.90"	27°52′52.92"	17	62.3	0.24	0.01
Tapia	TA	65°17′9.30"	26°35′42.90"	11	56.4	0.18	0.01

For each sampled tree, the spatial coordinates (altitude, latitude, and longitude) were recorded using a GPS device (Garmim^®^ eTrex).

The representative vouchers of each sampled tree were deposited at the SI Herbarium, Instituto de Botánica Darwinion, San Isidro, Buenos Aires, Argentina.

### AFLP methods and data analysis

#### DNA extraction

The DNA of young leaves was extracted with the DNeasy Plant Kit (QIAGEN Inc., Valencia, California, USA), following the manufacturer’s instructions. The DNA was stored at -20°C.

The AFLP assay was performed as described by Vos et al. [12], following the steps detailed in Pometti et al. [13]. Specifically, for the present work on selective amplification, four primer combinations were chosen: E + AAC/M + CTT (C2), E + AGG/M + CAG (C3), E + AAC/M + CAA (C4) and E +AAG/M + CAA (C5). In all cases, primers M + 3 were labelled with the fluorescent dye 6-FAM.

#### Data scoring and analysis

Each AFLP band was considered a single biallelic locus with an amplifiable (dominant) allele, scored (1), and a null (recessive) allele, scored (0).

The Bayesian likelihood method implemented in the program BAYESCAN v2.1 [14] was utilized to identify the existence of variants with non-neutral divergence among the six populations. The burn-in period was 50,000, the thinning interval was 10, the number of iterations was 100,000, the number of pilot runs was 20 and the length of each pilot run was 5,000. Therefore, all genetic diversity and population structure analyses were carried out on the set of putatively neutral loci.

To estimate the allele frequencies, the software AFLP-SURV [15] was employed using the Bayesian method with non-uniform prior distribution of allele frequencies, as described by Zhivotovsky [16], following Lynch and Milligan’s [17] approach. Nei’s [18] genetic diversity *H*_*E*_ and pairwise Nei’s [19] genetic distances between populations were also estimated using the software AFLP-SURV [15].

To estimate the distribution of genetic diversity, the analysis of molecular variance (AMOVA) was assessed by considering within-population and between-population components. The decomposition of variance by AMOVA was conducted following Excoffier et al. [20] while using the matrix approximations from Dyer et al. [21] with the software GeneticStudio [22]. Non-hierarchical Wright’s [23] *F*_*ST*_, was estimated with the package *HierFstat* [24, 25]. The significance of this estimate was obtained through a G test based on 5000 permutations.

To assess isolation by distance, a Mantel test was performed using the function “mantel.randtest” from the *ade4* package [25, 26], by testing the relationship between pairwise Nei´s [19] and geographic distances.

To identify the population genetic structure in the populations of *A*. *aroma*, two Bayesian model-based cluster analyses were contrasted: STRUCTURE [27] and Geneland [28]. The rationale for comparing these methods is that they might yield different results because they use different information (the former does not use geographical coordinates, whereas the latter does) and produce different visualizations of the putative distribution of the clusters detected. First, STRUCTURE version 2.3.4 [27] was used with a burn-in period and Markov chain Monte Carlo (MCMC) repetitions set to 50,000 and 100,000, respectively. The admixture model with correlated allele frequencies was selected; *K* was set at 1–8, and *K* values were averaged across 10 iterations. Using the rate of change in the log likelihood, the ad hoc statistic Δ*K* described by Evanno et al. [29] was estimated using STRUCTURE HARVESTER software [30]. The results from STRUCTURE were edited with the software CLUMPP 1.1.2 [31] and Distruct 1.1 [32] to obtain the plot.

The second method relied on the spatial cluster model implemented in the Geneland package [28] of the program R [25]. Following the user’s manual recommendations, the Markov chain Monte Carlo (MCMC) repetitions were set to 100,000, the thinning was set to 100 and the burn-in period was set to 100 (we eliminated the first 100 iterations when the curve was not constant); the number of groups (*K*) to be tested was set from 1–7. Each individual was assigned to one of *K* populations (1≤ *K*≤ 7) based on its multilocus genotypes and spatial coordinates. To confirm that the run was long enough, we obtained 10 different runs and compared the parameter estimates (*K*, individual population membership, maps). The best result was chosen based on the highest average posterior density.

To analyse the fine-scale spatial genetic structure (SGS), the approach described by Hardy [33] was utilized, studying each population through kinship coefficients (*F*_*ij*_). The assumed inbreeding coefficient was *F*_*IS*_ = 0.19, based on the average of *F*_*IS*_ estimates obtained for the same species from codominant allozyme markers [34]. The number of distance classes (distance intervals within which all pairs of sampling points are considered) was set to between 5 and 30 per population, in order to include at least 40 pairs of individuals in each distance class. To establish the relationship between geographic distance classes and genetic similarity, the regression slope of the kinship coefficients on log-transformed distances (*b*_*F*_) was estimated.

To determine the statistical significance of *F*_*1*_ (the mean kinship coefficient between individuals belonging to the first distance class) and the *b*_*F*_, the upper and lower bounds of the 95% confidence interval of *F*_*ij*_ were used, which were defined after 10,000 permutations of individuals within locations. The *Sp* statistic [3] was computed for each population based on the regression slope of kinship coefficients, as *Sp* = −*b*_*F*_/(1−*F*_*1*_). The *Sp* statistic was expected to summarize the intensity of SGS, allowing for a quantitative comparison among species and/or populations [3]. All estimations of SGS were performed using the software SPAGeDi v1.5 [35].

An indirect estimation of gene flow from the SGS estimates was performed assuming an equilibrium of isolation by distance in the fine-scale genetic structure. In such cases, the extent of gene flow can be expressed in terms of Wright’s neighbourhood size as *N*_*b*_≡*4πD*_*E*_*σ*_*g*_^*2*^, where D_E_ is the effective population density and *σ*_*g*_ is the mean-squared parent-offspring distance and can be estimated as the inverse of *Sp* [3, 36] yielding *N*_*b*_ = (*F*_*1*_−1)/*b*_*F*_. In this study, we estimated *N*_*b*_ and *σ*_*g*_ using the census density of populations and three predicted effective values (1/2, 1/4 and 1/10 of the census density).

## Results

In this study, a total of 852 AFLP bands in the interval of 50–400 bp were generated with the four primer pair combinations used. From these bands, 401 with total reproducibility were selected for all analyses to yield a 0% error rate.

The scan for *F*_*ST*_-outliers, conducted with BAYESCAN within the 401 AFLP loci (with a *q*-value threshold of 10%), did not detect selection for any locus.

The measurements of genetic diversity are summarized in Table 1. *H*_*E*_ varied from 0.18 in TA to 0.24 in SJ (mean *H*_*E*_ = 0.21), and *PPL* varied from 56.4% in TA to 67.1% in QU (mean *PPL* = 62.1%).

When analysing the components of genetic diversity with AFLP-SURV, the highest component was within populations (*Hw* = 0.21), while the between populations component (*Hb* = 0.07) was lower. The analysis of population structure by means of Wright’s [23] *F*_*ST*_ statistic (0.42) was highly significant (*P* = 5x10^-4^). When comparing pairwise Nei´s [19] and geographical distances matrices with the Mantel test, this result was not significant (*P* = 0.38).

The analysis of molecular variance indicated that the largest component of genetic diversity (60.7%) was found within populations and that the remaining (39.3%) was found between populations (Table 2).

**Table 2:** Analysis of molecular variance (AMOVA) based on 401 neutral AFLP loci in six populations of *A*. *aroma*. Φ = fixation index.

Source of variation	df	SSD	MSD	Variance (%)	ϕ	*p*
Between pop.	5	3683.96	736.79	39.3	0.393	0.001
Within pop.	164	6623.03	40.38	60.7		

Using the software STRUCTURE, a high peak of Δ*K* was found at *K* = 3, based on the AFLP dataset analysis determining the presence of three clusters. In this analysis, individuals from MI, RO, SJ and TA were grouped, as they were similar from a genetic perspective.

**Fig 2:**
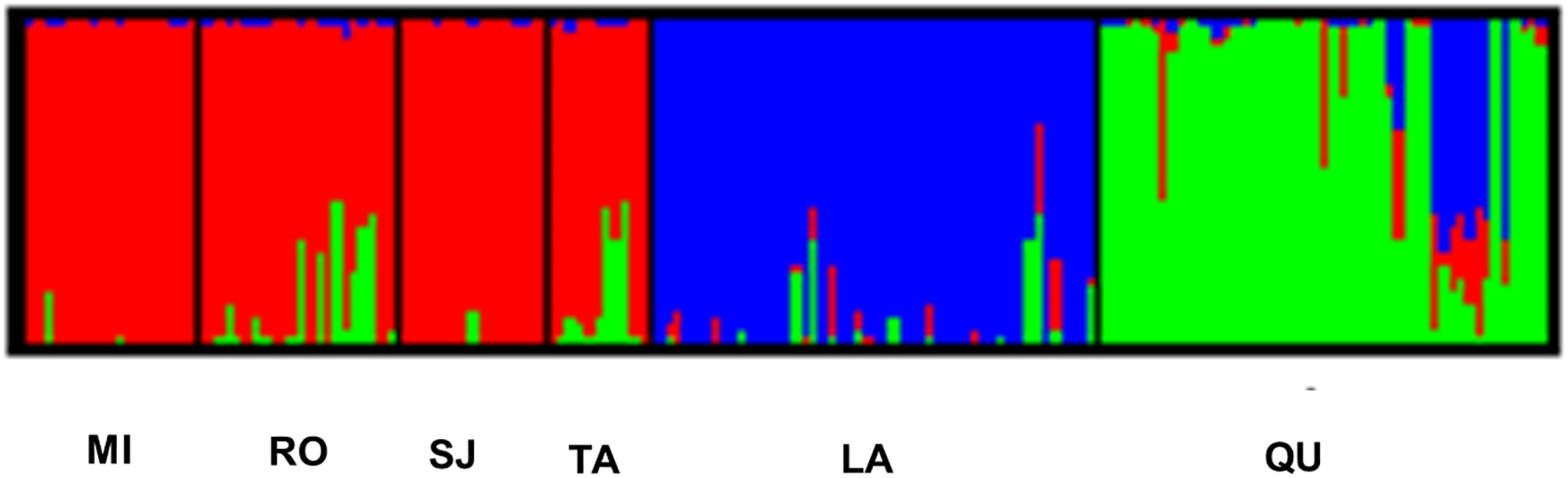
Clustering of individuals produced by STRUCTURE for *K* = 3. Each individual is represented by a vertical coloured line. The same colour for different individuals indicates that they belong to the same cluster or group. The population codes are the same as those shown in Fig 1.

The remaining two sample sites of *A*. *aroma*, LA and QU, constituted two differentiated clusters. Admixture was observed in all populations but was less prevalent in MI and SJ (Fig 2).

**Fig 3:**
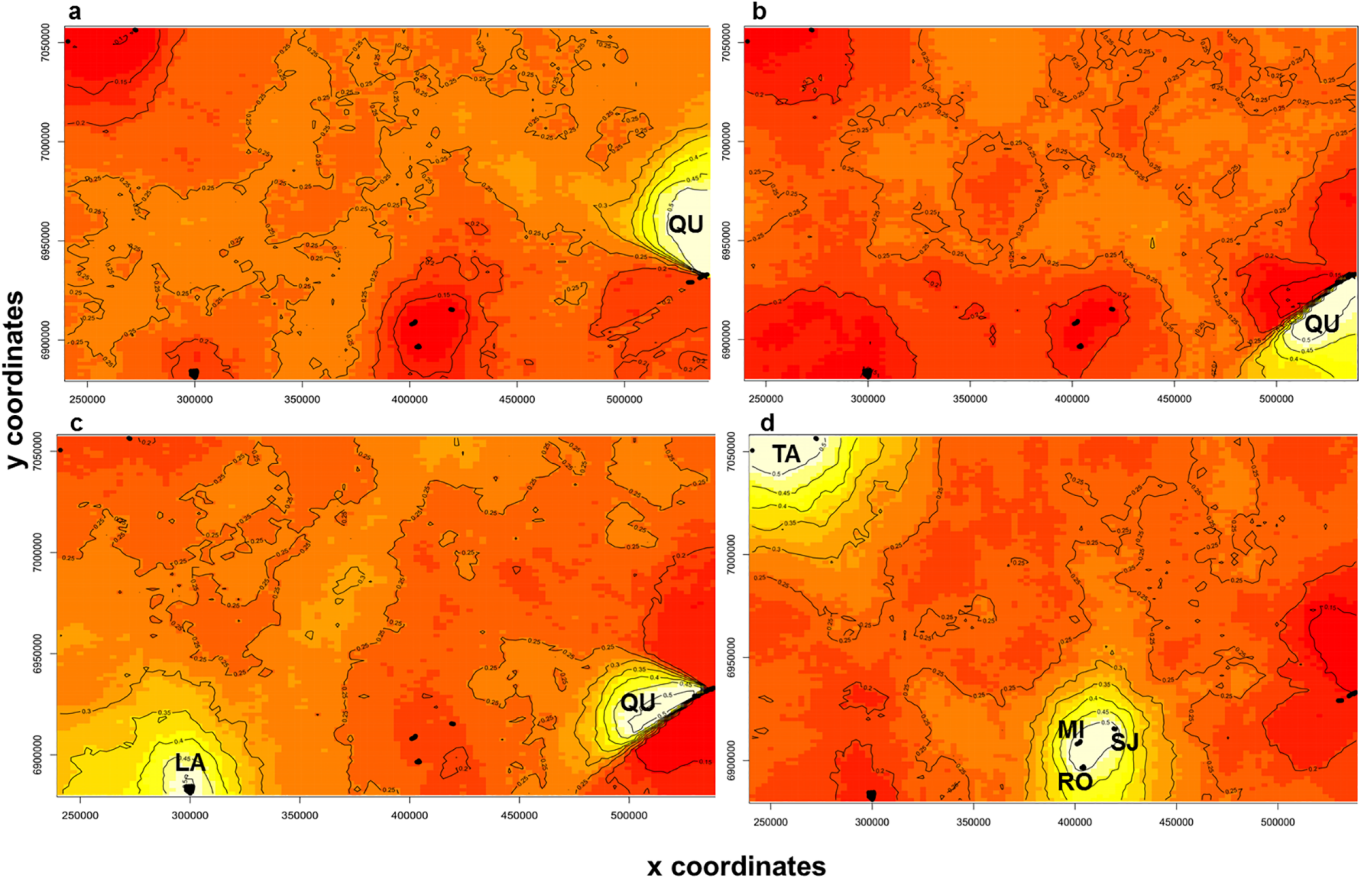
Spatial distribution of each group defined by Geneland at *K* = 4. (a) Cluster 1, (b) Cluster 2, (c) Cluster 3, and (d) Cluster 4. Clusters are indicated by areas with different intensities of colour. Lighter-coloured areas indicate a higher probability that individuals belong to that cluster. The population codes are the same as those shown in Fig 1.

Analysis using Geneland yielded a modal number of populations between 3 and 4, with a higher proportion of *K* = 4 (Table 3). The run with the highest average posterior density was selected. Clusters 1 and 2 included 33 and 10 individuals, respectively, from QU (Fig 3a and 3b). Cluster 3 was constituted by all individuals from LA and the remaining 7 individuals from QU (Fig 3c); and cluster 4 was composed of individuals from TA, RO, SJ and MI (Fig 3d).

**Table 3:** Multiple runs for inferring the number of populations with Geneland. Bold indicates the highest average posterior probability.

Run	Modal number	Mean of probability density	% of modal number
**1**	4	-50808.20	68.78
**2**	**4**	**-50200.06**	**69.33**
**3**	3	-53223.13	64.00
**4**	4	-51772.92	57.11
**5**	4	-50558.58	64.67
**6**	4	-50786.25	52.22
**7**	4	-50471.06	65.33
**8**	3	-52789.56	62.67
**9**	4	-51397.36	53.22
**10**	4	-51398.89	44.12

**Fig 4:**
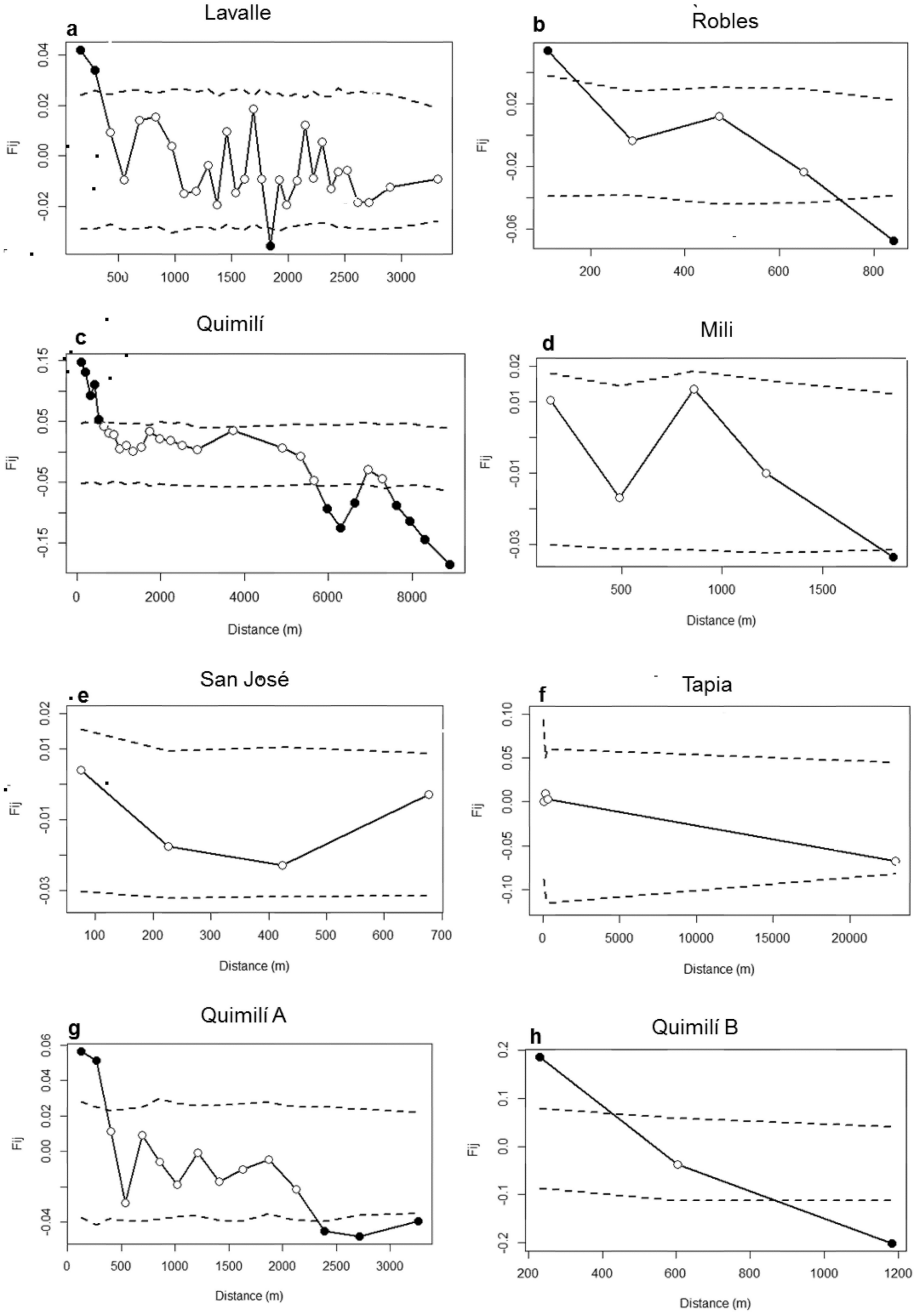
Correlograms showing the fine-scale spatial genetic structure (SGS) of the studied populations. (a) Lavalle, (b) Robles, (c) Quimilí, (d) Mili, (e) San José, (f) Tapia, (g) Quimilí A, and (h) Quimilí B. The filled circles indicate significant *F*_*ij*_ values. The dotted lines indicate the 95% confidence intervals (10,000 permutations).

Significant SGS was detected in short to medium distances in the LA, RO and QU populations (up to 530 m) (Fig 4a, 4b and 4c, respectively). However, no significant spatial genetic structure was detected in MI, SJ and TA (Fig 4d, 4e and 4f, respectively). In distance classes shorter than 1000 m, a pattern of positive *F*_*ij*_ was observed in almost all populations, and for larger distance classes, negative *F*_*ij*_ values were observed.

Populations QU and RO showed the strongest SGS and have a negative log slope (*b*_*F*_) (Table 4).

**Table 4:** Estimation of the fine-scale genetic structure at 401 AFLP markers in *A*. *aroma* populations. *F*_*1*_: multilocus kinship coefficient between individuals from the first distance class, *b*_*F*_: regression slope of *F* on natural log distance, *Sp*: quantification of the SGS, *N*_*b*_: neighbourhood size, *D*_*E*_: census density determined as trees/hectare (1 ha = 10,000 m^2^) and *σ*_*g*_: gene dispersal distance (in metres) using four estimates of effective densities (*D*_*E*_, *D*_*E*_*/2*, *D*_*E*_*/4* and *D*_*E*_*/10*). * *P*<0.01. The codes for populations are the same as shown in Table 1.

	*Sp*	*F*_*1*_	*b*_*F*_	*N*_*b*_	*D*_*E*_	*σ*_*g*_ *(D*_*E*_*)*	*σ*_*g*_ *(D*_*E*_*/2)*	*σ*_*g*_ *(D*_*E*_*/4)*	*σ*_*g*_ *(D*_*E*_*/10)*
**LA**	0.02	0.04	-0.01*	64.3	0.69	273.1	386.3	546.3	863.7
**MI**	0.01	0.01	-0.01	-	22.05	-	-	-	-
**RO**	0.05	0.05	-0.05*	20.5	8.19	44.6	63.1	89.2	141.1
**TA**	0.02	0.00	-0.02	-	50.00	-	-	-	-
**SJ**	0.01	0.00	-0.01	-	12.62	-	-	-	-
**QU**	0.07	0.15	-0.06*	15.2	2.72	66.7	94.3	133.3	210.8
**QUA**	0.03	0.06	-0.03*	31.9	3.40	86.4	122.1	172.7	273.1
**QUB**	0.23	0.14	-0.20*	4.4	2.03	41.4	58.5	82.8	130.9

The neighbourhood size was calculated for populations where SGS was significant and ranged from 15.2 in QU to 64.3 individuals in LA (Table 4).

The estimation of gene dispersal (*σ*_*g*_) was conducted considering four different effective densities in populations where SGS was significant. The extreme values of gene dispersal, which corresponded respectively to the highest and lowest density estimates in each population, were 273 and 864 m in LA, 45 and 141 m in RO, and 67 and 211 m in QU (Table 4).

Considering its patchy distribution, Quimilí (QU) was split into two groups of individuals: QUA (36 individuals) and QUB (14 individuals) (Fig 1). Significant SGS was detected in both groups (Table 3, Fig 4g and 4h), but differences were observed between the groups in both neighbourhood size and *Sp* estimates. The estimation of gene dispersal (*σ*_*g*_) ranged from 86 to 273 m in QUA and 41 to 131 m in QUB, depending on the value used for the estimated density.

## Discussion

Due to the importance of the *Acacia aroma* species in South America, in this study, we explored genetic diversity and population structure and characterized fine-scale spatial genetic structure of this species in Argentina. In this work, we observed high levels of genetic diversity, showing that populations of this species tend to maintain the majority of variability within populations, as do other species of *Acacia* and perennial woody outcrossed species [13, 37, 38, 39]. Additionally, significant SGS was detected in 3 of 6 populations.

Habitat fragmentation is a significant threat to the maintenance of biodiversity in many ecosystems. In general, the genetic consequences of habitat degradation focus on the reduced size of populations and increased spatial isolation of remnant populations. However, in some circumstances, fragmentation appears to increase gene flow among remnant populations, breaking down local genetic structure [6]. Therefore, it is important to study the genetic structure of populations when habitat degradation is occurring, in order to design management plans to maintain the genetic diversity of a species. The genetic diversity observed in *A*. *aroma* seemed to be relatively high when compared with other species sharing the same life history traits (citar Nybom 2004) and was similar to the values observed in other *Acacia* species such as *A*. *curvifructa* [37], *A*. *visco* [13] and *A*. *senegal* [40], which were also studied with dominant markers. Additionally, in this study, the determination of population structure was assessed with several approaches. Wright´s *F*_*ST*_ statistics (0.42) showed a strong signal of genetic structure among populations. However, the lack of significant correlation between geographic and genetic distance suggested that the differentiation among populations did not fit the isolation by distance model. This may be explained by assuming that gene flow is limited and that populations have not reached the migration-drift equilibrium [41]. This approach was consistent with the AMOVA results. Literature has established that long-lived and outcrossing species tend to maintain most of their variability within populations [42]. This is the case for *A*. *aroma*, that in this work showed that the majority of its molecular variance occurred within populations. This result also concurs with previous records for other South American and African species of *Acacia* [40, 43, 44, 45,].

The analysis with STRUCTURE showed that the optimal number of clusters (*K*) was 3. Conversely, Geneland analysis showed that the optimal number of clusters was *K* = 4, which shared a similar grouping to that of STRUCTURE but divided in two one of the clusters identified by the aforementioned program. In the two cases, populations of SJ, RO, MI and TA constituted a group. This finding could partially be due to the geographical proximity of SJ, RO and MI. Although TA is geographically farther in distance, there is a route (used by vehicles and pedestrians) that might promote gene flow (mediated by human activities) between TA and the first three populations, leading to the increased genetic similarity. The population of LA constituted a second cluster in STRUCTURE, with low levels of admixture. The individuals from QU constituted a third cluster, with evidence of admixture with a sizeable contribution from LA. This situation, with several differences, seems to be reflected in the plot from Geneland (Fig 3). In this case, the admixed individuals in QU were clustered with those from LA. The remaining individuals from QU were split into two clusters by Geneland.

Huang et al. [46] theorized that admixture, reflecting allele sharing, can result from incomplete lineage sorting of historically contiguous populations, which might be the case for LA and QU populations. The heterogeneity in the composition of QU, shown by Geneland, could be because this population occurs in a highly disturbed area, and the distribution of its individuals is patchy. Indeed, fragmentation and perturbation are expected to reduce the effective population size within patches and increase the genetic differentiation between the populations [6].

Vekemans and Hardy [3] noted that individuals located near each other tend to be more similar than those located farther apart. Additionally, isolation by distance modelling suggests that SGS is expected at the equilibrium between drift and dispersal. Therefore, limited gene dispersal may produce mating among related individuals and fine-scale spatial genetic structure. In the present study, the individuals who were geographically farther apart were revealed to have lower genetic similarity. Although the extent of gene dispersal (*σ*_*g*_) seems to be larger than the sampling area within sites, significant SGS was revealed in some populations, suggesting an isolation by distance pattern.

Previous research has demonstrated that SGS is correlated with the mating system, life history and population density [3]. Thus, the *Sp* statistic was proposed to synthesize SGS intensity and remains useful when comparing the strength of SGS in different populations. *A*. *aroma* is an outcrossing species, with its pollen and seed dispersed by animals. However, the mean *Sp* statistic estimated here (*Sp* = 0.057) was greater than the mean values presented by Vekemans and Hardy [3] for outcrossing species (*Sp* = 0.0126), species with animal-dispersed seeds (*Sp* = 0.0088), and species with pollen dispersed through animals (*Sp* = 0.0171). Additionally, this statistic was higher than the values observed for *Schinus molle* (*Sp* = 0.021) [47] and *Prosopis alba* (*Sp* = 0.003) [48] which are both species that share similar life history traits with *A*. *aroma*, since they are outcrosser trees with animals as the vectors responsible for seed and pollen dispersal. Spatial genetic structure can be affected by density, among other factors, because it can influence the rate of genetic drift [3]. In support of this concept, the three populations with the lowest densities, LA, RO and QU, are those which showed significant SGS. When splitting QU into two groups, thereby noting its patchy distribution, level of disturbance, geographical position of individuals and results obtained with Geneland, we observed a remarkably higher *Sp* value in QUB (0.23) than in the QU sample as a whole (0.07) and in QUA (0.03). The differences observed between the two groups could be partially attributed to the low level of disturbance in QUB. This group of individuals, although small, is situated in a more pristine and unmanaged area than those from QUA. Since the six populations studied here presented different levels of disturbance and ecological characteristics, our work suggests the need to evaluate the ecological aspects of the life history and landscape for each population.

When comparing the estimates of gene dispersal, we obtained similar values for the four possible densities considered here as those estimated with AFLP markers in other outcrossers, animal, pollen and seed dispersed trees, like *Chrysophyllum sanguinolentum*, *Eperua grandiflora* and *Virola michelii* [49]. Our results on high gene dispersal are consistent with the basis that *A*. *aroma* is a species pollinated by bees, with seeds dispersed by livestock and large mammals. Additionally, the distance of gene dispersal is usually inversely related to population density [3], and this occurred in LA, RO and QU, where the density was lower than 9 individuals per ha. Curtu et al. [50] proposed that low density can act as a barrier to pollen and seed dispersal, thereby yielding SGS.

## Conclusions

When aiming to design conservation, management, and sustainable use strategies for a species, it is important to understand the patterns of genetic diversity [42, 51, 52]. In the Chaco Region of Northwest Argentina, the contributing factors to environmental degradation have been deforestation, uncontrolled firewood harvesting, livestock overstocking, and in some areas, tillage of non-arable lands [11]. Analysis of population genetic structures shows that managers should consider either three or four (depending on analysis) genetically distinct groups when making management decisions. Our work describes, for the first time, the SGS and gene dispersal parameters in *A*. *aroma*, which are valuable for the management and conservation of this and other *Acacia* species. The current SGS analysis provides information that may be used during sampling of individuals and seed collection for *ex situ* conservation and reforestation programmes. Our findings suggest that for *A*. *aroma* reforestation and management programmes, sampling should consider a minimal distance of 50 to 150 m to minimize genetic relatedness among sampled seeds in each area. In cases of disturbed populations with low density such as LA, the minimum and maximum distances should be much greater (270 and 870 m, respectively). In summary, the findings of this study are important for managing and conserving the extant trees and populations of *A*. *aroma* in the fragmented landscapes, such as Lavalle and Quimilí, and provide baseline information on the spatial structuring and dispersal of genes in this species. The combination of molecular markers and robust statistical approaches constitutes an effective strategy for supporting programmes that mitigate the deterioration and desertification of semi-arid lands in Argentina.

## Supporting information

S1 Table Allele frequencies for the 401 AFLP loci analysed in *Acacia aroma* populations. Sample size (N); frequency of the AFLP fragment or marker (freq_frag); estimated frequency of the null allele (freq_-all); estimated variance of the frequency of the null allele (var_-all).
(XLSX)
